# More Children Means More Tumours? We Can Do More to Protect the Health of Our Kids—A Call for a New Epidemiology That Can Change the World

**DOI:** 10.3390/epidemiologia4020018

**Published:** 2023-06-01

**Authors:** Prisco Piscitelli, Alessandro Miani, Enrico Greco, Alessandro Distante, Francesco Schittulli, Adele Civino

**Affiliations:** 1 Department of Biological and Environmental Sciences and Technologies (DISTEBA), University of Salento, 73100 Lecce, Italy; 2 Euro Mediterranean Scientific Biomedical Institute (ISBEM), Rue de Bellard 20, 1040 Bruxelles, Belgium; 3 Italian Society of Environmental Medicine (SIMA), 20123 Milan, Italy; alessandro.miani@gmail.com (A.M.); enrico.greco@units.it (E.G.); 4 Department of Environmental Science and Policy, University of Milan, 20149 Milan, Italy; 5 Euro Mediterranean Scientific Biomedical Institute (ISBEM), 72023 Mesagne, Italy; distante@isbem.it; 6 Italian League for Fighting Against Cancer (LILT), 00162 Rome, Italy; f.schittulli@lilt.it; 7 Local Health Authority ASL Lecce, 73100 Lecce, Italy; adelecivino@gmail.com

In encyclopaedic dictionaries published until 1955, the word “tumour” was defined as an “occupational disease suffered by the workers of chemical industries”, thus referring to a very specific cause [1]. This clear reference to a chemical-related aetiology of cancer progressively disappeared in subsequent decades, opening the door to generic explanations based on a multifactorial pathogenesis. Today, cancer is generally associated with population ageing as a consequence of random (stochastic) accumulation of oxidative genetic damage, highlighted by an ongoing improvement in our diagnostic capacities. However, this explanation does not clarifies why the highest and more rapid increases in cancer incidences (average annual variations) are observed in the youngest age groups, including children, who are not exposed to traditional risk factors such as cigarette smoking (“children do not smoke”), occupational triggers, or to prolonged “unhealthy lifestyles”. Similarly, the incredibly high numbers of the current “cancer pandemic” cannot be reasonably explained only by the increased diagnostic capacities of modern medicine [2].

The ACCIS project (Automated Childhood Cancer Information System) conducted by the International Agency for Research on Cancer (IARC) on 63 huge cancer registries in 19 European countries has highlighted an annual increase up to 1.5% in all paediatric cancers and +2% in the first year of life, being the most significant increases related to lymphomas, sarcomas, germ-cell and nervous system tumours [2,3,4,5]. New annual cases of paediatric cancers have been estimated to rise up to 13.7 million globally from 2020 to 2050, resulting in 9.3 million deaths among children living in low-income countries [6]. Cancer has become the primary disease-related cause of death in all paediatric age groups in Europe in the last 20 years [2].

In this frame, it is unreasonable to minimize this phenomenon when assessing the paediatric cancer incidence at national level by simply reporting that the observed increases are consistent with the current international trends, which are known to be continuously rising. We cannot resign ourselves to the “axiom” that regions with higher numbers of children must also experience a higher incidence of paediatric cancers. When did we start thinking that more children should mean more tumours? The answer from the medical community cannot be limited to count the number of deaths and incident cases, usually providing these data after many years, so that they can be less useful for health policy makers.

The increase in cancer incidence in the first year of life has been possibly linked to trans-placental (maternal–foetal) exposure to pro-carcinogenic agents or transgenerational transmission of epigenetic marks in gametes as a consequence of exposure to different environmental contaminants in the “first thousand days of life” or during fertile adult years, thus calling for a shift in the etiological paradigm of carcinogenesis theory [7,8]. Actually, in children we cannot assume—as in adults and elderly people—a progressive accumulation of casual (stochastic) DNA mutations, as presumed by the currently accepted pathogenic model (namely, the so-called “somatic mutation theory”, SMT). Recognizing the most appropriate etiological theory for carcinogenesis would allow us to implement proper primary preventive measures, such as removing individual exposure to chemicals and environmental carcinogens (IARC 1 and 2). We are not protecting the health of our children if we cannot even begin to address the issue of cancer incidence reduction in the right way: children should not suffer from tumours. The fact that few voices of condemnation have been heard concerning the unacceptability of this dramatic phenomenon is probably related to the need to change our current social and economic models. **Public health policies should be focused on keeping people healthy (true primary prevention) and removing the causes of cancer**.

As part of the medical and scientific community, we have the duty to primarily contribute to a decisive change of direction, able to safeguard the next generations’ health with the same efforts we are putting into climate change research. It may be that we must stop “the count” and discussions on the consistency between “observed” and “expected” rates. We should simply stop “expecting” to observe a persistent increase in paediatric cancer cases or we fail in our primary mission: providing decision makers with evidence and data upon which they have enough information to protect people’s health, paying particular attention and priority to children.

Information concerning the main causes of hospitalizations and deaths stratified per age group and geographical area should be provided to decision makers almost in real time—within a few months—by using the large available datasets of Healthcare Systems or those of insurance companies. Data can be easily obtained about all the different types of cancers from 2000 to date. **Modern medicine should be focused on primary prevention**. Specific preventive actions must be adopted based on risk factors which characterize populations at the local level, also taking into account social determinants of health, as fighting against social inequalities can contribute to prevent diseases and cancers.

We ask our colleagues worldwide to come together and address the challenge of a new vision of medicine and epidemiology which is able to change the world. The statistics that we are generating must turn into preventive actions by promptly highlighting the emerging threats to people’s health—with a special focus on children—in order to provide possible solutions, starting from remarking the unacceptability of having cancer as the primary disease-related cause of death in our children. To accomplish this goal, epidemiological observations (which represent the “facts”) should guide the development of consistent etiological theories for cancer, as well as for all the other conditions which are also dramatically increasing in paediatric ages, such as congenital malformations, autoimmune and metabolic diseases (including type 1 diabetes) or neurodevelopmental disorders (i.e., autism), taking into account the interactions between epigenome, lifestyle and environment. This will ultimately turn into unexpected opportunities both for pharmacological and non-pharmacological primary prevention in order to reduce the burden of the new epidemics of the XXI century.
